# Dimensions of Hybrid and Nanohybrid Mouthguards for Mixed Martial Arts Fighters–Evaluation of a New Method of Fabrication

**DOI:** 10.3390/polym14245369

**Published:** 2022-12-08

**Authors:** Katarzyna Mańka-Malara, Maciej Trzaskowski, Elżbieta Mierzwińska-Nastalska

**Affiliations:** Department of Prosthodontics, Medical University of Warsaw, 02-097 Warsaw, Poland

**Keywords:** injury prevention, nanoparticles, combat sports, composites, mouthguard thinning, dentistry, dental materials, protective splint, centric relation, prosthodontics

## Abstract

Ethylene vinyl acetate mouthguards are the most often used custom protective intraoral appliances by combat sports practitioners. However, due to the difficulties in maintaining the hygiene of such mouthguards and thinning during fabrication, resulting in difficulty in predicting final dimensions, they may not be the optimal solution. The aim of this research was to evaluate an innovative method of mouthguard formation using intraoral modeling of the mouthguard pattern, hybrid acrylic material, and the addition of ZnO nanoparticles. Seventeen mouthguards patterns and 34 custom mouthguards were evaluated: 17 hybrid and 17 nanohybrid. A total of 1122 measurements were performed: each mouthguard and pattern was measured at 22 points. Statistical analyses were performed with the use of IBM^®^ SPSS^®^ Statistics 27.0.0 software (IBM, Armonk, NY USA). The mean thickness of the patterns and mouthguards at all labial areas of central incisors were between 4.65 and 4.80 mm. The thickness at the buccal surface of the first molar was between 3.71 and 4 mm, and at the occlusal surface between 3.40 and 3.56 mm in the cusp area. All measurements of hybrid and nanohybrid mouthguards were strongly and highly correlated with the measurements of the mouthguard patterns. Hybrid and nanohybrid mouthguards are an advantageous alternative to thermoformed custom appliances.

## 1. Introduction

Mouthguards should have favorable mechanical properties and adequate dimensions, so as to be well adapted to the oral cavity structures. The material most thoroughly studied for their fabrication is ethylene-vinyl acetate copolymer (EVA), from which both “boil and bite” and custom mouthguards can be made [1,2,3,4]. This type of appliance is the gold standard in the fabrication of protective splints. However, EVA copolymers are sensitive to repeated exposure to high temperatures and prolonged heating. A study by Gould et al. [5], determining the thermal properties of mouthguards using differential scanning calorimetry and dynamic mechanical analysis, showed significant limitations of flexible polymeric materials when used in wide temperature ranges. When the glass transition temperature is reached below the oral cavity temperature, insufficient impact energy damping properties can occur [5]. The issue of proper shaping after thermal plasticization applies both to “boil and bite” and custom thermoformed splints. Researchers have noted the problem of obtaining the optimal thickness on all tooth surfaces, which is necessary to provide the proper protection against the effects of injury [6,7,8,9,10]. After plasticizing at high temperature, the material is stretched and thinned [11]. In the literature, a loss of up to 25% of the thickness of the protector on the occlusal surface and 50% on the labial surface has been described, resulting in a reduction in ability to reduce impact forces [12]. Recently, numerous authors have published recommendations for the proper fabrication of a thermoformed protective splint, looking for a method of fabrication that prevents the loss of material thickness [6,7,8,10,11].

There have also been reports that the surface of thermoformed EVA is uneven and may become a problem with longer-term use, as it is easily destroyed during sanitization procedures [13]. Some authors have presented attempts to introduce prevention of bacterial contamination by coating EVA with nanoparticles of chlorhexidine compounds gradually released from the material [14]. Nagai et al. [15] proposed introducing a bioactive filler into ethylene vinyl acetate of pre-reacted glass-ionomer (S–PRG). Yoshida et al. [16] described the introduction of silver nanoparticles into an EVA mouthguard. In prosthodontics, the influence of incorporation of ZnO nanoparticles in the denture base material was described [17,18,19]. Such additions to the mouthguard material could result in better hygienic properties of the final splint. Currently, new techniques and materials are being introduced into mouthguard fabrication [20,21,22]. Splints made of hybrid acrylic have been shown to have favorable mechanical characteristics [23], proper functionality of the finished mouthguard splint [2,24], and the possibility of regular disinfection by the user [13]. The aim of the conducted research was to compare the dimensions of hybrid and nanohybrid mouthguards, measured on the labial surface of the central incisor, and buccal and occlusal surface on the first permanent molar, and to evaluate the predictability of custom mouthguard fabrication with the use of a customized impression tray, by comparing the dimensions of the final appliances with the directly modelled mouthguard patterns.

## 2. Materials and Methods

### 2.1. Custom Mouthguard Fabrication

**Figure 1:**
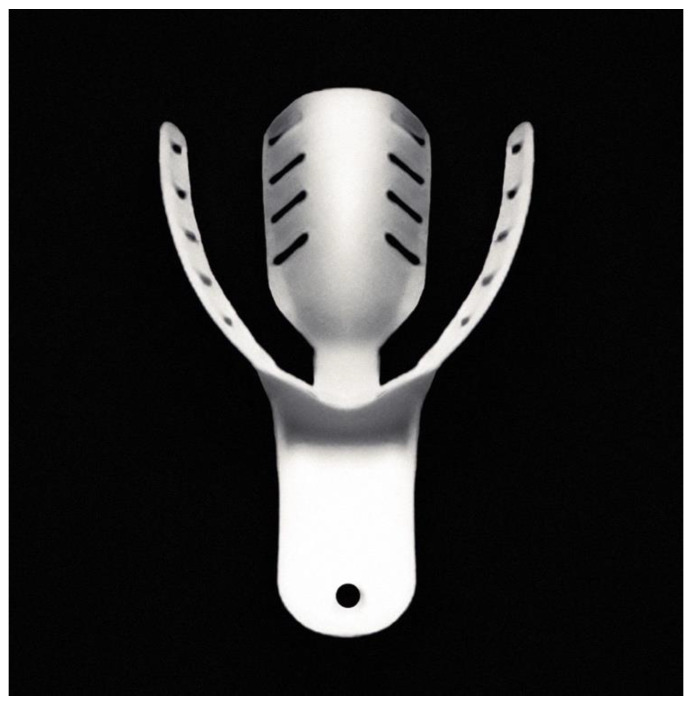
Customized impression tray for hybrid mouthguard fabrication.

**Figure 2:**
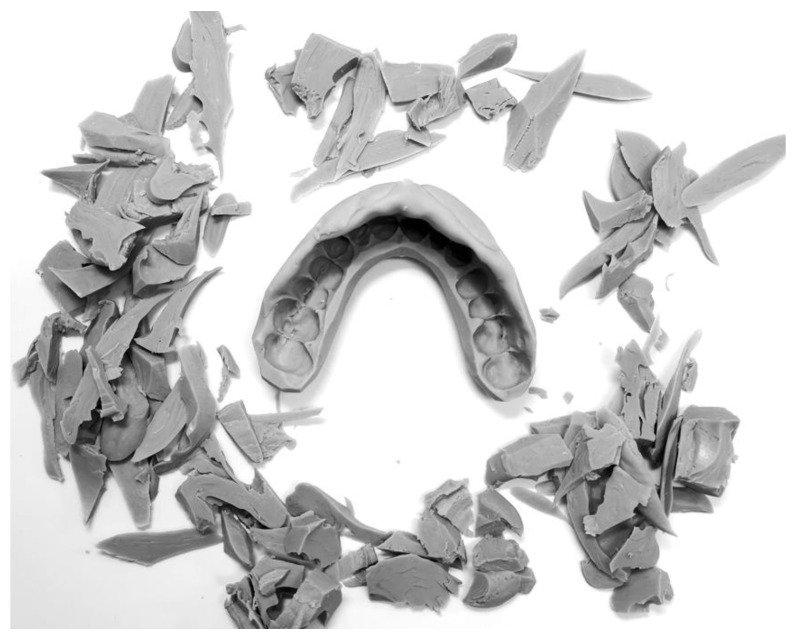
Silicone mouthguard pattern during adjustment using a scalpel.

This study evaluated 34 custom protective splints: 17 hybrid mouthguards and 17 nanohybrid mouthguards with zinc oxide nanoparticles, as well as 17 mouthguard patterns made by intraoral modeling [25]. They were made for athletes professionally training in mixed martial arts (MMA). The study was conducted with the approval of the Medical University of Warsaw Bioethics Committee–KB/7/A2020. For each participant, a thorough medical and dental examination was performed, with particular emphasis on the evaluation of the stomatognathic system. A customized impression tray, without the occlusal surface, apart from the occlusal stop between the incisal region, was used (Figure 1). An appropriate size of tray was selected, to cover the entire maxillary dental arch, leaving room for the impression material. Before forming the mouthguard pattern, the ability to close the mouth to the desired distance with simultaneous control of the mandibular movement, and response to the command to stop the movement of joining the dental arches were verified. Using the occlusal stop in the incisal area, the repeatability of the movement was verified, and the mouthguard could be formed with a centric relation. Zeta Plus (Zhermack, Badia Polesine, Italy) was used to make the mouthguard pattern. The impression of teeth and alveolar processes of the maxilla, and the correct contact of the maxillary and mandibular teeth in the centric relation, were recorded simultaneously. The mouthguard pattern was shaped using a scalpel (Figure 2).

Two custom mouthguards were made for each fighter: a hybrid mouthguard using hybrid acrylic material (Impak, Vernon–Benshoff Comp., Albany, NY, USA), and a nanohybrid mouthguard made of hybrid acrylic with zinc oxide nanoparticles (ZnO NPs). To obtain hybrid splints made of Impak material, the mouthguard pattern was placed in a polymerization can. Once the gypsum had set, the silicone material was removed and Impak material, mixed in a 1.3:1 ratio of powder and liquid, was put in its place. The correct composition is essential for optimal flexibility of the finished splint; if a larger volume of liquid is used, the final appliance is more flexible after polymerization. For the nanohybrid mouthguards, ZnO nanopowder obtained using microwave solvothermal synthesis, composed of single spherical crystallites, and having skeleton density 5.24 ± 0.05 g/cm^3^, specific surface area 39 ± 1 m^2^/g, average particle size 30 ± 0.1 nm, and average crystallite size (from Scherrer’s formula) 22–25 nm, prepared for the previous research [17,18,19], was used. It was suspended in liquid monomer of a resin. An appropriate amount of resin powder was added, so that a final concentration of 7.5% ZnO NPs could be obtained [17,18,19]. After compression and polymerization, according to the manufacturer’s recommendations, for 5 h at a temperature of about 74 °C, both types of mouthguards were cleaned and polished in a dental laboratory. The mouthguard pattern was used twice, the same for both protectors. The sequence of flasking was random, to avoid the influence of the order. Only mouthguards, where the mouthguard patterns were not damaged after first removal and could be used for second splint fabrication were used in the presented evaluation.

### 2.2. Measurements of Mouthguards and Pattern Dimensions

**Figure 3:**
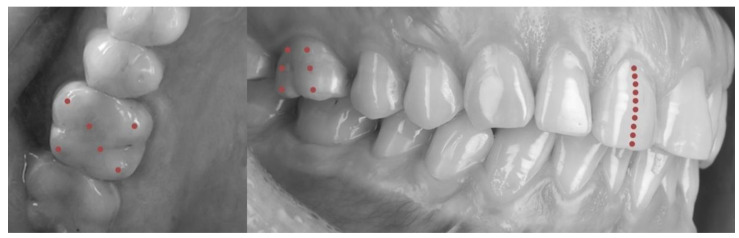
Measurement points for the evaluation of mouthguard thickness.

Mouthguards and pattern thicknesses were measured using a digital measuring device (Digital Caliper, Falab, Gdynia, Poland) having a resolution accuracy of 0.01 mm and measuring range of 0–9.5 ± 0.5 mm. The device did not have a spring, to prevent potential distortion [26]. A total of 1122 measurements were performed, and each mouthguard and pattern was measured at 22 points [8]. The precise locations of the measurement points are presented in Figure 3. The thickness at the surface of the central incisors was measured at 10 points evenly spaced along the center of the tooth, from the incisal edge to the cervical margin. The thickness at the buccal surface of the first molar was measured at six points: on the mesiobuccal and the distobuccal cusp, on the center under these cusps, and in the cervical area. The thickness on the occlusal surface of the first molar was measured at six points: the mesiobuccal cusp, the mesiolingual cusp, the distobuccal cusp, the distolingual cusp, the mesial fovea, and the distal fovea.

### 2.3. Statistical Analysis

Statistical analyses were performed with the use of IBM^®^ SPSS^®^ Statistics 27.0.0 software (IBM, Armonk, NY, USA), and all graphs were prepared with Microsoft Excel. There were two types of analyses conducted: a comparison of the dependent variables, to evaluate whether dimensions of the pattern were recreated in the final appliances; and the calculation of mean values and standard deviations of specific areas of the mouthguard, to compare the achieved dimensions with those achieved in thermoformed appliances described in the literature [8]. To verify whether the two types of mouthguards differed significantly from the pattern, a series of t-tests for dependent variables were conducted. As the number of analyses was high, a Bonferroni correction for multiple comparison was used. All equivalent measurement points from the pair pattern–hybrid mouthguards, and the pair pattern–nanohybrid mouthguards, were compared. In the analyses, we considered *p*–values < 0.05 as significant. The mean thickness and standard deviation were calculated for each area of the appliance. Ten points from the area of central incisors were grouped in into incisal (three points from the incisal edge), central (four points in the central area), and cervical areas (three points above the cervical line). The six points from the buccal surface of the first molar were also grouped into cusp (the mesiobuccal and distobuccal cusp), central (two central points), and cervical (two cervical points). The points from the occlusal surface were grouped into the cusp area (mesiobuccal cusp, mesiolingual cusp, distobuccal cusp, and distolingual cusp) and the fovea area (mesial and distal fovea).

## 3. Results

**Figure 4:**
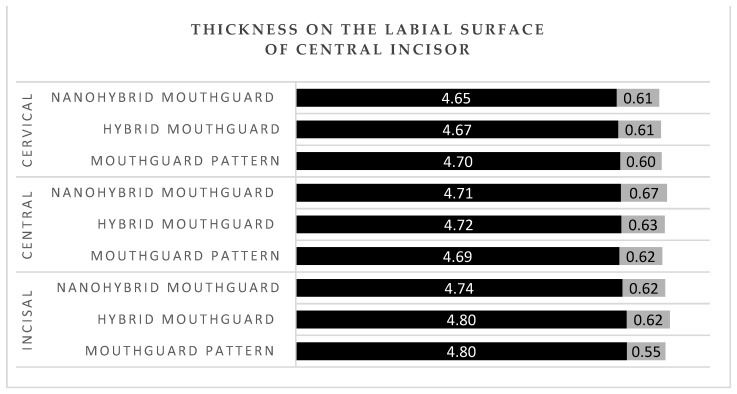
Thickness at the labial surface of the central incisor. Black—mean value; grey—standard deviation (SD).

**Figure 5:**
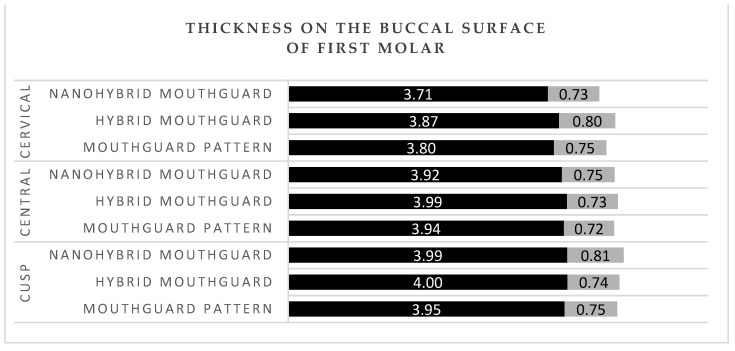
Thickness at the buccal surface of first molar. Black—mean value; grey—standard deviation (SD).

**Figure 6:**
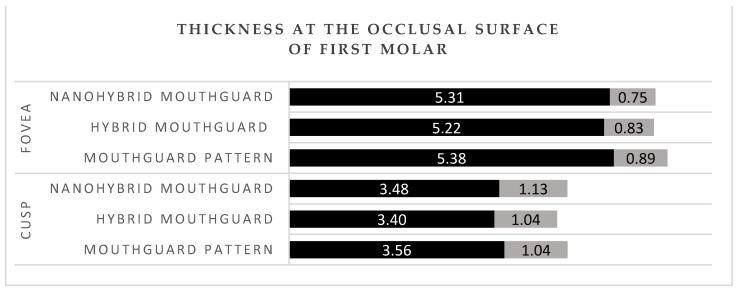
Thickness at the occlusal surface of first molar. Black—mean value; grey—standard deviation (SD).

The mean thickness of the patterns and mouthguards at all labial areas of the central incisors (incisal, central, and cervical) was between 4.65 and 4.80 mm (Figure 4). The thickness of the patterns and mouthguards at the buccal surface of first molar was between 3.71 and 4 mm (Figure 5). The mean thickness of the patterns and mouthguards at the occlusal surface of first molar was between 3.40 and 3.56 mm in the cusp area 5.22, and 5.38 in the fovea area (Figure 6).

All measurements of hybrid mouthguards were strongly and highly correlated to the measurements of the mouthguard patterns (Table 1). Relatively low but still strong and significant correlation coefficients were observed for the distobuccal cusp and mesial fovea. There were no significant differences in the average values of the hybrid mouthguard and mouthguard pattern measurements (i.e., all uncorrected *p*–values were above 0.05).

In the case of the nanohybrid mouthguards, there were strong and high correlations between the measurements and the measurements of the mouthguard pattern for all variables, despite the distobuccal cusp having a moderately strong correlation (Table 2). There were no statistically significant differences between the measurements and the pattern.

## 4. Discussion

The use of intraoral modeling of the protector pattern is simple to perform and can be successfully applied clinically. It provides an opportunity for an initial intraoral verification of the appliance size, at the stage of mouthguard pattern. The described method of hybrid and nanohybrid mouthguard fabrication provides highly predictable dimensions of the protective splint, even in the case of incorporating ZnO nanoparticles into the material. There were no statistically significant differences between the mouthguards and patterns, and thus it can be concluded that after initial verification of the pattern dimensions during adjustment, the achieved dimensions were replicated in the final appliance. To compare, according to Farrington et. al. [27], the overall EVA material thinning during fabrication is 59.5%. In addition, the introduction of a modification that enables the fabrication of an intraoral protector in a centric relation, with modeled occlusal contacts, allows for the fabrication of a restoration that may be optimal in terms of the stomatognathic system. The study by Verissimo et al. [28] emphasized that achieving balanced occlusion and maximum contacts with the teeth results in the proper stress distribution during impaction, through proper stabilization of the protective splint. Other innovative mouthguard designs have also been described in the literature. Westerman et al. [29] recommended introducing air into the EVA material, which resulted in a 32% reduction in the imparted impact forces. However, the addition of a foaming agent to this material did not result in improved protective properties [30]. The possibility of using relining materials to adjust the intraoral protective splint after they have become deformed or after changes have occurred in the user’s dentition, without affecting their mechanical properties, has been evaluated by other researchers [31]. McClelland et al. [32] described raising the labial edge of the protector within the oral vestibule by 2 mm, an even distribution of occlusal contacts, rounded cheek-contact parts for the protective splint, and narrow palatal edges, to improve user comfort. Other researchers also recommended a significant reduction in the palatal portion of the intraoral protector, without significantly compromising retention [33]. Modification of protective splints is likely to be even greater in the future. In addition to changes in manufacturing, improvements in materials and adjustments to improve functional properties, the literature includes reports of embedded sensors continuously testing for the presence of selected compounds in the user’s saliva. Kim et al. [34] described a wireless biosensor that assesses the uric acid content of saliva, integrated into the intraoral protector.

In professional MMA, fighters use mouthguards as the only protection in the head area. Intraoral protective splints are made of a material with energy-absorbing properties. There are no conclusive reports on the reduction of concussion risk with the use of mouthguards, but biomechanical studies have shown a significant reduction in transmitted force to the central nervous system with the use of protective splints [35,36,37]. Among those who sustained an injury, the damage assessed on a symptomatic scale was statistically significantly more severe among those who trained without a mouthguard [38,39]. Tribst J.P.M et al. [40] performed impact simulations using three-dimensional finite element analysis in different skeletal classes. They showed that the presence of a mouthguard during impact caused a decrease in stress magnitudes in the condyle and the articular disc. In MMA, proper thickness of the final splint is especially important, as training in this discipline entails a high exposure to injuries and head trauma [41]. Application of a hybrid mouthguard, where the accurate thickness can be planned for each athlete individually and maintained during the fabrication procedure, would be beneficial for this discipline. Research conducted on laminated mouthguards using the same measurement points and calculations of mean values for the same area showed that the thickness at the labial surface of central incisors was between 2.7 and 2.85 mm in the incisal and the central area, and 2.85 and 3 mm in the cervical area [8]. In the current study, the mean obtained thicknesses were 4.8 mm in hybrid mouthguards and 4.74 mm in nanohybrid mouthguards in the incisal region, 4.72 mm (hybrid), and 4.71 mm (nanohybrid) in the central area, and 4.67 mm (hybrid) and 4.65 mm (nanohybrid) in the cervical area. In the buccal region of the first molar, laminated mouthguards had mean thickness of 2.8 mm in the cusp area, between 2.8 and 2.9 mm in the central area, and between 2.9 and 3.1 mm in the cervical area [8]. Hybrid and nanohybrid mouthguards had respectively 4 mm and 3.99 mm in the cusp area, 3.99 mm and 3.92 mm in the central area, and 3.87 mm and 3.71 mm in the cervical area. According to literature, the optimal thickness at labial and buccal surface for a mouthguard is 4 mm; with EVA material having a thickness of 3 mm transmitting more than twice the force that was passed through the 4 mm material when impacted with the same force [42]. Hybrid acrylic material has also proven to have more favorable energy absorption properties than ethylene vinyl acetate [4,23]. In disciplines with a higher exposure to maxillofacial trauma, the use of hybrid mouthguards ensures better protection for the athlete.

According to Glass et al. [43], a mouthguard should be perceived as a therapeutic device and be replaced after every change of its surface. In a microbiological analysis of protective splints used by 62 American football players, 81 pads isolated 356 bacterial strains, 22 yeast-like fungi, and 107 molds [44]. Due to the risk of cross infection, some researchers even suggest that a mouthguard should be disposed of after a single use [45]. Many patients do not maintain sufficient oral hygiene: 85.5% of athletes choose tap water rinsing as the main method of mouthguard cleaning [46,47]. The disinfection recommended in the literature is currently used by only 2.7% of mouthguards users [46]. ZnO nanoparticles have already been successfully introduced into denture bases, having advantageous antibacterial and antifungal properties, with no cytotoxic effects on host cells [17,18,19,48]. Their addition to mouthguard materials could reduce the risk of infection and the negative effects of protective splint application on the health of users.

The major limitation of the current study was that the measurements were only made on selected points on the central incisor, the first molar, and occlusal surface of the first molar. There is a possibility that such an appliance does not have adequate dimensions for all teeth, especially if the user has misaligned teeth. However, the described methodology was used in many articles described in the literature, and therefore was chosen as the most reliable to replicate and compare the achieved results. Further prospective studies are also needed to verify the mechanical and clinical characteristics of such appliances over longer observation periods. It could be also discussed whether the adaptation of the mouthguard pattern relies on dentists’ experience and be time-consuming. However, for patients and athletes requiring significant protection, and at the same time maintaining the optimal occlusal contacts and function of the stomatognatic system, this still is a recommended method.

## 5. Conclusions

Hybrid and nanohybrid mouthguards are an advantageous alternative to thermoformed custom appliances, due to the possibility of adding nanoparticles to the hybrid acrylic material and for fabricating a mouthguard in centric relation. The dimensions of the final appliances prepared using direct fabrication of the mouthguard pattern on the customized impression tray were highly correlated with the models; therefore, such prepared appliances have an appropriate thickness and can be used as protection from the impact forces in different sport disciplines. The individually planned dimensions of the protective splint are an especially advantageous feature for mixed martial arts competitors, where the exposure to head trauma is high. The evaluated method of mouthguard fabrication is predictable and can be recommended for clinical use.

## Figures and Tables

**Table 1:** Correlations between dimensions of the mouthguard patterns and hybrid mouthguards.

Compared Variable	M Pattern	SD Pattern	M Hybrid	SD Hybrid	T	Uncorrected *p*–Value	Corrected *p*–Values	Correlation	Correlation *p*
1 labial incisal	4.79	0.57	4.82	0.64	–0.83	0.42	1	0.96	<0.001
2 labial incisal	4.81	0.57	4.77	0.64	0.90	0.38	1	0.97	<0.001
3 labial incisal	4.81	0.55	4.80	0.62	0.28	0.78	1	0.96	<0.001
4 labial center	4.79	0.69	4.75	0.68	0.74	0.47	1	0.96	<0.001
5 labial center	4.65	0.71	4.72	0.67	–1.32	0.21	1	0.96	<0.001
6 labial center	4.67	0.58	4.71	0.63	–0.79	0.44	1	0.95	<0.001
7 labial center	4.65	0.53	4.71	0.56	–1.32	0.21	1	0.96	<0.001
8 labial cervical	4.68	0.59	4.71	0.63	–0.50	0.63	1	0.94	<0.001
9 labial cervical	4.75	0.64	4.68	0.62	1.23	0.24	1	0.94	<0.001
10 labial cervical	4.68	0.59	4.64	0.63	0.63	0.54	1	0.90	<0.001
mesial cusp	3.87	0.73	3.89	0.73	–0.65	0.52	1	0.98	<0.001
mesial cusp center	3.86	0.72	3.92	0.73	–0.94	0.36	1	0.93	<0.001
mesial cusp cervical	3.69	0.78	3.75	0.81	–1.12	0.28	1	0.96	<0.001
distal cusp	4.04	0.78	4.10	0.76	–1.03	0.32	1	0.95	<0.001
distal cusp center	4.03	0.72	4.05	0.74	–0.37	0.71	1	0.94	<0.001
distal cusp cervical	3.91	0.72	3.99	0.79	–1.29	0.22	1	0.95	<0.001
mesiobuccal cusp	3.75	1.21	3.58	1.21	1.41	0.18	1	0.91	<0.001
mesiolingual cusp	3.42	0.82	3.39	1.00	0.20	0.85	1	0.86	<0.001
distobuccal cusp	3.52	0.97	3.41	0.97	0.58	0.57	1	0.64	0.01
distolingual cusp	3.56	1.20	3.23	1.02	1.90	0.08	1	0.79	<0.001
mesial fovea	5.55	0.89	5.30	0.77	1.49	0.16	1	0.65	0.00
distal fovea	5.21	0.87	5.13	0.90	0.67	0.51	1	0.86	<0.001

**Table 2:** Correlations between dimensions of mouthguard patterns and nanohybrid mouthguards.

Compared Variable	M Pattern	SD Pattern	M Nanohybrid	SD Nanohybrid	T	Uncorrected *p*–Value	Corrected *p*–Values	Correlation	Correlation *p*
1 labial incisal	4.79	0.57	4.76	0.63	0.36	0.36	1	0.90	<0.001
2 labial incisal	4.81	0.57	4.73	0.63	1.19	0.13	1	0.91	<0.001
3 labial incisal	4.81	0.55	4.72	0.62	1.57	0.07	1	0.92	<0.001
4 labial center	4.79	0.69	4.74	0.68	1.09	0.15	1	0.97	<0.001
5 labial center	4.65	0.71	4.70	0.73	–0.87	0.20	1	0.96	<0.001
6 labial center	4.67	0.58	4.70	0.67	–0.40	0.35	1	0.91	<0.001
7 labial center	4.65	0.53	4.72	0.63	–0.64	0.27	1	0.77	<0.001
8 labial cervical	4.68	0.59	4.71	0.59	–0.52	0.31	1	0.95	<0.001
9 labial cervical	4.75	0.64	4.67	0.57	1.33	0.10	1	0.94	<0.001
10 labial cervical	4.68	0.59	4.58	0.69	1.02	0.16	1	0.82	<0.001
mesial cusp	3.87	0.73	3.87	0.79	–0.08	0.47	1	0.96	<0.001
mesial cusp center	3.86	0.72	3.88	0.71	–0.46	0.33	1	0.96	<0.001
mesial cusp cervical	3.69	0.78	3.67	0.71	0.19	0.43	1	0.89	<0.001
distal cusp	4.04	0.78	4.11	0.83	–0.64	0.27	1	0.84	<0.001
distal cusp center	4.03	0.72	3.95	0.80	0.71	0.24	1	0.84	<0.001
distal cusp cervical	3.91	0.72	3.76	0.78	1.17	0.13	1	0.73	<0.001
mesiobuccal cusp	3.75	1.21	3.67	1.39	0.50	0.31	1	0.89	<0.001
mesiolingual cusp	3.42	0.82	3.39	1.03	0.19	0.43	1	0.79	<0.001
distobuccal cusp	3.52	0.97	3.52	1.02	0.02	0.49	1	0.49	0.05
distolingual cusp	3.56	1.20	3.34	1.10	1.62	0.06	1	0.88	<0.001
mesial fovea	5.55	0.89	5.37	0.70	1.38	0.09	1	0.79	<0.001
distal fovea	5.21	0.87	5.26	0.82	–0.52	0.31	1	0.89	<0.001

## Data Availability

The data presented in the study are available on reasonable request from authors of this article.
